# Design of Low-Cost and Highly Energy-Efficient Convolutional Neural Networks Based on Deterministic Encoding

**DOI:** 10.3390/s25103127

**Published:** 2025-05-15

**Authors:** Tiance Tong, Qiang He, Xiaofei Nie, Yudi Zhao

**Affiliations:** School of Information & Communication Engineering, Beijing Information Science and Technology University, Beijing 102206, China; 2022020500@bistu.edu.cn (T.T.); qiang.he@bistu.edu.cn (Q.H.); 2023020521@bistu.edu.cn (X.N.)

**Keywords:** stochastic computing, deterministic encoding, neural networks, image recognition

## Abstract

Stochastic Computing has attracted extensive attention in the deployment of neural networks at the edge due to its low hardware cost and high fault tolerance. However, traditional stochastic computing requires a long random bit stream to achieve sufficient numerical precision. The long bit stream, in turn, increases the network inference time, hardware cost, and power consumption, which limits its application in executing tasks such as handwritten recognition, speech recognition, image processing, and image classification at the near-sensor end. To realize high-energy-efficiency and low-cost hardware neural networks at the near-sensor end, a hardware optimization design of convolutional neural networks based on the hybrid encoding of deterministic encoding and binary encoding is proposed. By transforming the output signals from the sensor into deterministic encoding and co-optimizing the network training process, a low-cost and high-energy-efficiency convolution operation network is achieved with a shorter bit stream input. This network can achieve good recognition performance with an extremely short bit stream, significantly reducing the system’s latency and energy consumption. Compared with traditional stochastic computing networks, this network shortens the bit stream length by 64 times without affecting the recognition rate, achieving a recognition rate of 99% with a 2-bit input. Compared with the traditional 2-bit stochastic computing scheme, the area is reduced by 44.98%, the power consumption is reduced by 60.47%, and the energy efficiency is increased by 12 times. Compared with the traditional 256-bit stochastic computing scheme, the area is reduced by 82.87%, and the energy efficiency is increased by 1947 times. These comparative results demonstrate that this work has significant advantages in executing tasks such as image classification at the near-sensor end and edge devices.

## 1. Introduction

With the rapid development of technologies such as the Internet of Things (IoT) and autonomous driving, the number of sensors used to capture images, audio, and other information has increased dramatically. The vast amounts of raw data collected by these sensors contain a significant amount of unstructured and redundant information, leading to severe challenges in energy consumption, response time, data storage, and communication bandwidth during subsequent information processing [1]. To alleviate the data transmission burden on sensor nodes, deploying certain image processing tasks at the near-sensor edge can significantly reduce latency and redundant data storage in the sensing-to-computation process, thereby improving system real-time performance and energy efficiency [2]. Meanwhile, convolutional neural networks (CNNs) have been widely applied in critical fields such as indoor positioning [3], intrusion detection [4], malware identification [5], edge computing, and artificial intelligence [6]. These application scenarios impose stringent requirements on computational efficiency and hardware costs, particularly when deployed on resource-constrained edge devices. However, neural networks typically require complex circuit structures to perform a large number of multiplication and addition operations, which demand extensive logic gates and hardware resources in traditional binary computing paradigms [7]. Consequently, executing neural networks using binary computing results in high power consumption, posing a significant challenge for mobile and edge computing devices.

**Figure 1:**
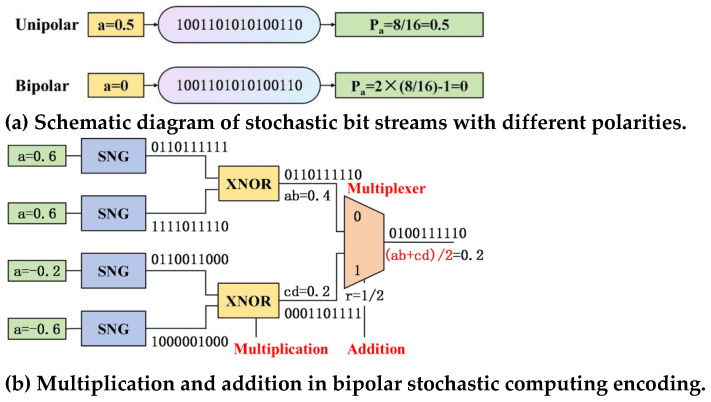
Schematic Diagram of Stochastic Computing.

To address the performance and energy efficiency challenges of neural network computation architectures, it is essential to explore new information processing paradigms beyond traditional high-precision digital computing. Stochastic Computing (SC) is a fault-tolerant computing paradigm that utilizes a Stochastic Number Generator (SNG) to produce bit streams encoded in a stochastic manner. Using simple logic gates, SC performs operations such as multiplication and summation, with precision improving as the bit stream length increases [8]. Stochastic bit streams are sequences where each bit carries equal weight, and binary numbers can be mapped to stochastic bit streams using either unipolar or bipolar encoding methods [9], as shown in Figure 1a. In the unipolar encoding scheme, the probability of each bit being set to “1” is proportional to the represented value a. In the bipolar scheme, the bit stream represents values within the range [−1,1], where the probability of each bit being set to “1” is proportional to (a + 1)/2. A bit stream of length n contains several “1”s ranging from [0,n], allowing it to represent n + 1 different values. Since the bipolar scheme enables the representation of both positive and negative values, it is more suitable for neural networks.

Compared to binary computing, stochastic computing offers the following advantages in neural network applications:

(1) Stochastic computing performs complex arithmetic operations using simple logic gates, reducing the required number of logic components, thereby simplifying hardware design and lowering computational power consumption and hardware costs [10]. For example, multiplication, which is a complex operation in traditional binary computing, can be implemented with a single AND gate in stochastic computing, while addition can be represented using a multiplexer (MUX), as shown in Figure 1b. Simple circuits are particularly important for battery-powered mobile and edge computing devices.

(2) Since each bit in a stochastic bit stream has the same weight, it has high fault tolerance, and single-bit errors do not significantly impact the overall result as in binary computing [11].

(3) When using bipolar encoding, the input and output values of stochastic computing are both within the range [−1, 1], avoiding the need for additional normalization operations.

**Figure 2:**
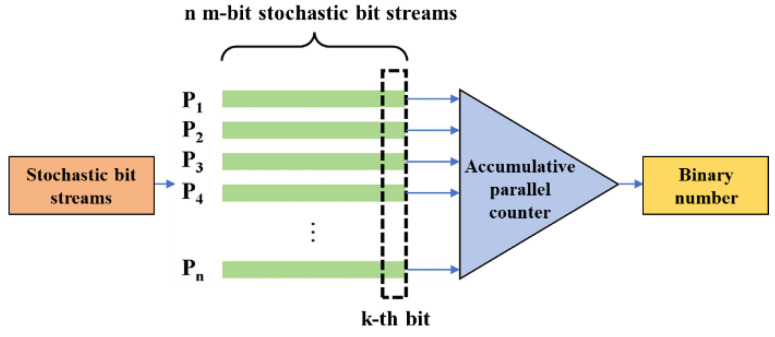
Accumulative parallel counter.

However, traditional stochastic computing-based neural networks have certain limitations. First, stochastic computing requires long bit streams to maintain computational accuracy, resulting in prolonged computational delays. Second, most existing stochastic computing methods rely on a large number of pseudo-random number generators to produce stochastic bit streams, which severely restricts circuit area and energy efficiency [12]. Additionally, when using bipolar encoding, traditional stochastic computing exhibits low multiplication accuracy for near-zero data, which contradicts the sparsity characteristic of neural networks [13]. Furthermore, neural networks frequently use Accumulative Parallel Counters (APC) for stochastic addition, as shown in Figure 2 [14]. APC is an electronic counter used for counting and accumulating the number of occurrences of events. Based on the principle of parallel counting, it can process multiple input signals simultaneously, enabling efficient and rapid accumulation. In stochastic addition, APC obtains the sum of the k-th bit from n m-bit stochastic bit streams using a parallel counter, stores the result in a register, and ultimately accumulates all bits from the n m-bit stochastic bit streams to produce a binary-form sum. However, using APC for addition introduces additional stochastic computing and binary conversion circuitry, significantly increasing circuit area. Finally, due to the distribution characteristics of convolutional kernels in neural networks, representing them with short-bit streams remains challenging.

To address these issues, this paper proposes a convolutional neural network hardware optimization design based on deterministic encoding. By converting image sensor output signals into deterministically encoded bit streams, the first convolutional layer of the network achieves low-cost and high-energy-efficiency multiply–accumulate operations with shorter bit streams. Additionally, a co-optimization of the network training process is conducted for the hybrid convolutional neural network combining deterministic and binary encoding. The optimized network achieves superior image classification performance even with extremely short bit streams, significantly reducing system latency and energy consumption.

This paper proposes a hardware optimization design for convolutional neural networks that leverages a hybrid deterministic and binary encoding scheme, achieving high energy efficiency and low cost through the use of an extremely short bitstream. Additionally, a co-optimization training algorithm for the first convolutional layer is developed, which supports 2-bit deterministic encoding while maintaining a recognition accuracy of 99%.

The remainder of this paper is organized as follows: Section 2 provides a review of related work on stochastic computing and its applications in neural networks. Section 3 describes the proposed method in detail, including the near-sensor deterministic encoding approach and the hybrid encoding neural network design. Section 4 presents the experimental results and analysis, demonstrating the effectiveness of the proposed method in terms of hardware efficiency and fault tolerance. Finally, Section 5 concludes the paper and outlines future research directions.

## 2. Related Work

Stochastic computing, due to its neuron-like information encoding method, can significantly reduce hardware requirements and energy consumption in neural network systems. Currently, stochastic computing has been successfully applied to various neural networks, achieving progress in multiple aspects. Zhe Li et al. [15] proposed a joint optimization method for components in deep stochastic convolutional neural networks to ensure high computational accuracy. By modifying the original network structure to simplify stochastic computing hardware design, the optimized feature extraction block improved computation accuracy by 42.22% compared to the non-optimized version, and the network’s test error rate was reduced to 3.48% from 27.83%. Mohamad Hasani Sadi et al. [16] introduced an efficient deep convolutional neural network inference framework based on stochastic computing. They designed a novel approximate parallel counter using multiplexers (MUX) to reduce input data and improved the stochastic Rectified Linear Unit (ReLU) activation function, making it more closely resemble the actual ReLU function while being compatible with APC-based adders. Additionally, they proposed a new method to implement the Softmax function in the stochastic domain with minimal resources, reducing hardware area, delay, and power consumption. These optimizations reduced hardware area overhead by approximately 17% and path delay by about 18% when implementing the LeNet-5 network. Sunny Bodiwala et al. [17] proposed an efficient deep neural network accelerator based on stochastic computing, optimizing its activation function. This work developed an improved stochastic computing neuron architecture for deep neural network training and implementation. By integrating stochastic computing, the method significantly reduced hardware occupation while maintaining high scalability. Experimental results showed that this approach improved classification accuracy on the Modified National Institute of Standards and Technology database (MNIST) by 9.47% compared to traditional binary computing methods. This research provides an effective solution for deploying complex deep-learning models on resource-constrained devices. M. Nobari et al. [18] proposed an efficient Field-Programmable Gate Array (FPGA)-based implementation of deep neural networks using stochastic computing, addressing the slow convergence speed and high hardware resource consumption of traditional SC methods. By limiting stochastic bit stream length and establishing precise synchronization among processing units, convergence time was significantly reduced. Additionally, the study proposed a novel probability estimator architecture that eliminated feedback loops in traditional probability estimators, improving convergence speed and accuracy. Implemented on the Xilinx FPGA Virtex-7 chip using Verilog hardware description language, experimental results demonstrated that this method reduced hardware resource utilization by over 82%, lowered power consumption, and increased deep neural network accuracy by 2%. This research provides an effective solution for implementing efficient deep-learning models on resource-constrained hardware platforms. Junxiu Liu et al. [19] proposed a stochastic computing-based hardware spiking neural network system for implementing pairwise spike-timing-dependent plasticity. By leveraging stochastic computing technology, the study simplified multipliers, adders, and subtractors in traditional hardware spiking neural networks, significantly reducing hardware resource consumption. Experimental results showed that, compared to existing technologies, this system reduced hardware resource consumption by 58.0%, with register usage decreasing by 65.6%. Tianmu Li et al. [20] introduced a new method called range-extended stochastic computing to improve the accuracy and efficiency of stochastic computing in neural network accelerators. By enhancing OR-gate-based stochastic computing accumulation functions, this method increased computational precision while maintaining performance advantages, achieving a 2-fold reduction in bit stream length at the same precision and improving energy efficiency by 3.6 times compared to traditional binary addition. Furthermore, the study proposed an optimized training method that incorporated bit stream computation simulation, activation calibration, and error injection, accelerating the training speed of range-extended stochastic computing neural networks by 22 times, making training on large datasets such as ImageNet feasible. Huiyi Gu et al. [21] proposed a novel computing-in-memory architecture based on magnetic random-access memory for Bayesian neural networks using stochastic computing. This architecture aimed to address the high computational complexity and extensive sampling operations required for deploying traditional Bayesian neural networks on edge devices. By performing computations directly within memory and leveraging the unique properties of magnetic random-access memory, an efficient computing-in-memory approach was realized. Neural network parameters were represented as bit streams, and by redesigning the computing-in-memory architecture, reliance on complex peripheral circuits was reduced. Additionally, a real-time Gaussian random number generator was designed using the random switching properties of magnetic random-access memory, further improving energy efficiency. Evaluations using Cadence Virtuoso Analog Design Environment showed that, while maintaining accuracy, this architecture reduced energy consumption by over 93.6% compared to traditional FPGA-based von Neumann architectures. This work offers an effective solution for deploying Bayesian neural networks on resource-constrained edge devices. K. Chen et al. [22] proposed a stochastic computing-based artificial neural network architecture design incorporating a novel unscaled adder and input data preprocessing method to enhance hardware reliability and computational accuracy. The adder combined a T-Flipflop adder and a finite-state machine linear gain unit, eliminating scaling effects in stochastic computing and generating accurate sums. Experimental results indicated that compared to traditional neural network designs, this architecture reduced power consumption and area costs by 48–81% and 51–92%, respectively. This research provides an effective solution for achieving high-reliability, high-accuracy neural networks in edge computing scenarios with resource constraints.

Table 1 presents the main contributions and data comparisons of the aforementioned references.

In summary, a series of studies have demonstrated that the stochastic computing paradigm can significantly reduce the hardware requirements and energy consumption of neural network systems, presenting broad development prospects. However, all the aforementioned studies face a common challenge: achieving high computational accuracy with stochastic computing typically requires long bit streams. This means that complex computations involve processing a large volume of bit stream data, leading to increased computational latency and resource consumption. Additionally, stochastic computing relies on a substantial number of random number generators to produce stochastic bit streams, which occupy extra hardware resources and increase circuit area and complexity.

**Figure 3:**
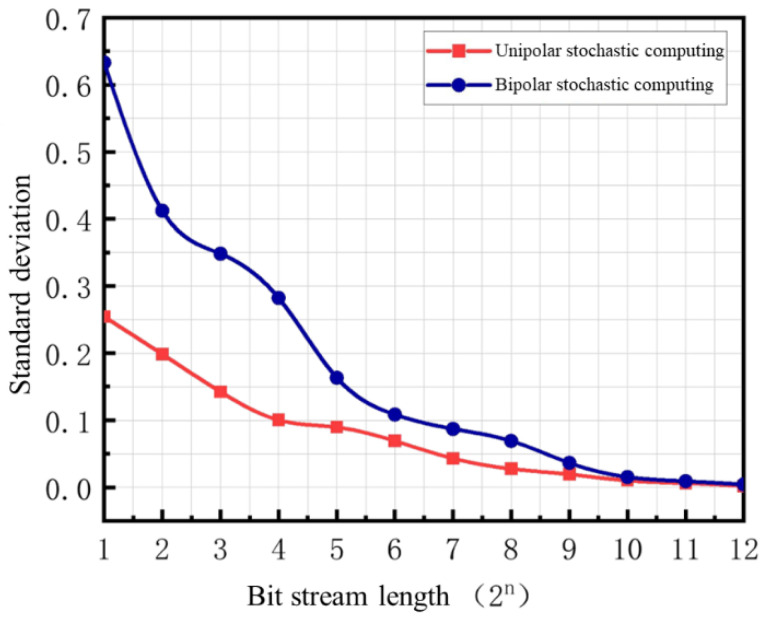
Standard Deviation of Stochastic Computing Multiplication-Accumulation Results from Binary Computing under Different Bit Stream Lengths.

To verify the relationship between the accuracy of stochastic computing multiplications and additions and the length of the bit stream, Figure 3 presents a 5 × 5 convolution kernel using both unipolar and bipolar encoding methods for multiplication, with accumulation performed via APC adder circuits. The results are compared with the binary results (all binary values used in this paper are full-precision binary). As shown in the figure, the accuracy of multiplication–accumulation computations depends on the length of the input stochastic bit stream—longer bit streams yield more accurate results. Under the same bit stream length, unipolar stochastic computing exhibits a lower standard deviation from binary computation results than bipolar stochastic computing. This is because bipolar stochastic computing represents values in the range of [−1, 1], doubling the range of unipolar stochastic computing [0, 1], but at the cost of reduced resolution, leading to lower accuracy compared to unipolar encoding of the same length.

While increasing the bit stream length improves computational accuracy, it also introduces significant computational latency and energy consumption issues. To address bit stream correlation problems, J. Alspector et al. [23] attempted to generate uncorrelated bit streams using multiple pseudo-random sources, but their approach still required substantial hardware resources. Gschwind, Michael et al. [24] reduced the number of required random sources by encoding one of the stochastic bit streams deterministically during multiplication; however, their method still consumed considerable hardware resources. Therefore, how to leverage the advantages of low-cost stochastic computing circuits in hardware neural networks while reducing the cost and energy consumption of input bit stream generation and achieving high computational accuracy with shorter input bit streams are the key to the edge-side high-efficiency neural network circuit design in this paper.

## 3. Method

### 3.1. Near-Sensor Deterministic Encoding

**Figure 4:**
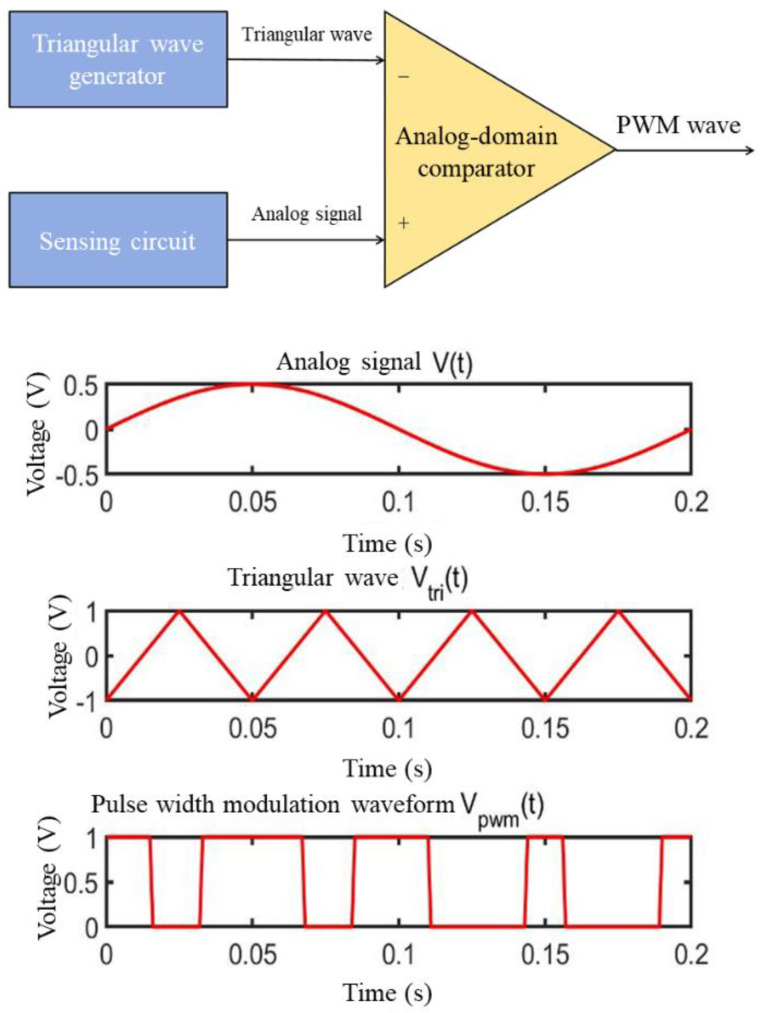
Generation of PWM (Pulse Width Modulation) waves.

For tasks such as image classification, pixel information must first be acquired at the image sensor before being processed by a neural network. A complementary metal-oxide-semiconductor (CMOS) image sensor converts the perceived light intensity into an electrical signal proportional to the light intensity through photodiodes in its pixels. These electrons are collected, integrated, amplified, and read out as an analog voltage at the end of the exposure process. Finally, an analog-to-digital converter (ADC) converts the voltage into a digital signal [25]. In this process, conventional binary-encoded neural networks require an ADC circuit, which entails significant hardware overhead to convert light intensity into digital signals. Traditional stochastic-encoded neural networks further require the digital signals to be transformed into stochastic bit streams via a stochastic number generator (SNG) [26]. To avoid the substantial resource consumption and delay associated with converting data from the analog to the digital domain, the proposed approach directly converts the analog voltage data obtained by the sensor into a time-domain data encoding format for the neural network input layer, thereby eliminating the need for additional ADC and SNG circuits. Figure 4 illustrates the process of converting the analog voltage signals acquired by the image sensor into time-domain signals. The circuit consists of a ramp generator and an analog-domain comparator. By comparing the analog voltage signal with a triangular wave using the analog-domain comparator, a pulse width modulation (PWM) signal is generated [27]. Through this process, pixel value information is encoded as the fraction of time the signal remains high (ON) relative to the low (OFF) state in each cycle. The duty cycle of the PWM signal corresponds to the analog voltage level output by the sensor: a higher voltage level results in a wider PWM pulse, representing a larger pixel value. The voltage range of the triangular wave is determined by the range of the analog voltage signal. For instance, if the analog voltage falls between the minimum and maximum values of the triangular wave, the circuit produces a PWM signal with a 50% duty cycle, corresponding to 0.5 in unipolar bit streams and 0 in bipolar bit streams. Thus, PWM signals with different duty cycles can serve as inputs to stochastic computing circuits. However, since “1”s and “0”s in PWM signals appear in clusters rather than randomly, these inputs no longer constitute stochastic bit streams but instead form deterministic bit streams [28]. A PWM-based deterministic bit stream of length *n* contains between 0 and n occurrences of “1”, allowing it to represent n + 1 distinct values.

To align with the PWM-type pixel value input and improve the computational accuracy of deterministic random computation, the weight values of the neural network will adopt a shift-uniform deterministic encoding scheme [29], further reducing the use of SNG. By decomposing the binary weights into a polynomial form, each term’s coefficient is encoded as a bit stream with a uniform distribution of 0s and 1s, followed by the corresponding shifts. Finally, the pre-generated encodings are summed. The specific method is as follows:

In the deterministic bit stream, the “1” of the input 1 is placed at the front of the bit stream, as shown in Equation (1):

(1) $$A^{L}\left(t\right)=\left\{\begin{array}{c} 1,t=1,2,3,\ldots ,A\\ 0,t=A+1,\ldots ,L \end{array}\right.$$

where *A* is the number of “1”s in the bit stream, and *L* is the total length of the bit stream.

For another input 2, the positions of the “1”s are given as shown in Equation (2):

(2) $$C^{L}\left(t\right)=\left\{\begin{array}{c} 1,t=round((L/C)\cdot m)+1\\ 0,others \end{array}\right.$$

where *C* is the number of “1”s in the bit stream, *L* is the total length of the bit stream, *m* = 0, 1, 2, …, *C* − 1, which is used to denote the periodicity, and round denotes rounding.

Thus, the binary number *C* can be expressed as shown in Equation (3):

(3) $$C=c_{k-1}s\left(2^{k-1}\right)+c_{k-2}s\left(2^{k-2}\right)+...+c_{0}s\left(2^{0}\right)$$

The part 2^i^ in the formula can be expressed as Equation (4):

(4) $$C_{2^{i}}^{L}\left(t\right)=\left\{\begin{array}{c} 1,t=m\cdot 2^{k-i}+1\to t\mathrm{mod}2=1\\ 0,others \end{array}\right.$$

Thus, the bit stream *C^L^* can be expressed as the sum of $C_{2^{0}}^{L}$ to $C_{2^{k-1}}^{L}$, which is represented by Equation (5):

(5) $$\begin{array}{cc} c_{k-1}s\left(2^{k-1}\right) & 10101010101010101010...\times c_{k-1}\\ + & +\\ c_{k-1}s\left(2^{k-2}\right) & 10001000100010001000...\times c_{k-2}\\ + & +\\ c_{k-3}s\left(2^{k-3}\right) & 10001000100010001000...\times c_{k-3}\\ + & +\\ \vdots & \vdots \\ + & +\\ c_{0}s\left(2^{0}\right) & 10000000000000000000...\times c_{0}\\ ↓ & ↓\\ C & C^{L} \end{array}$$

To merge all substreams into a single bit stream, we right-shift each substream by *T* bits, where *T* = 2^k−i−1^ − 1.

Equation (6) demonstrates an example of deterministic encoding with a bit stream length of 16 bits:

(6) $$\begin{array}{cc} C_{2^{3}}\gg 0 & 1010101010101010\times c_{3}\\ C_{2^{3}}\gg 1 & 0100010001000100\times c_{2}\\ C_{2^{3}}\gg 3 & 0001000000010000\times c_{1}\\ C_{2^{3}}\gg 7 & 0000000100000000\times c_{0}\\ C & c_{3}c_{2}c_{3}c_{1}c_{3}c_{2}c_{3}c_{0}c_{3}c_{2}c_{3}c_{1}c_{3}c_{2}c_{3}0 \end{array}$$

**Figure 5:**
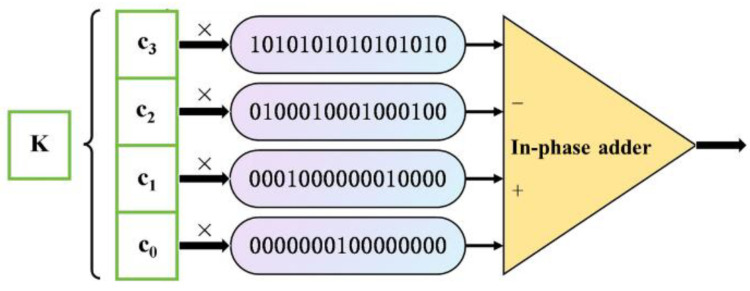
Shift Uniform Deterministic Encoding Circuit.

As shown in Figure 5, *K* represents the number of ones in the bit stream when deterministically encoding numbers within the range of [−1, 1], with c_0_, c_1_, c_2_, and c_3_ being the binary digits of K. Since the bit stream length in Equation (6) is 16 bits, the range of K is [0, 15], and the number of binary digits is 4. These binary digits are multiplied by the corresponding shift-uniform deterministic bit stream signals, and the multiple input signals are added together and output as a composite signal using an in-phase adder. In this encoding method, the number of ones in a bit stream of length n ranges from [0, n − 1], and a total of n distinct values can be represented.

### 3.2. Hybrid Encoding Neural Network Design

**Figure 6:**
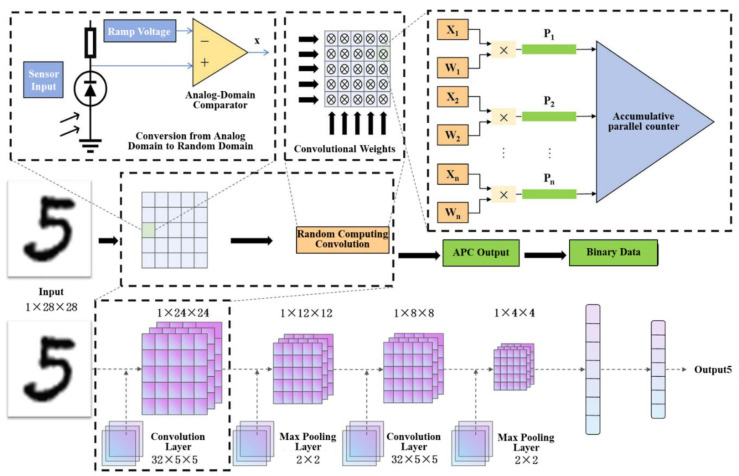
Schematic Diagram of Convolutional Neural Networks Combining Stochastic Computing and Binary Computation.

Convolutional Neural Networks (CNNs) are a type of deep learning model particularly suited for processing grid-structured data, such as images. By mimicking the working principles of the human visual system, CNNs can automatically and hierarchically extract image features [30]. LeNet-5 is a classic convolutional neural network (CNN) [31] with a simple yet effective structure. It consists of an input layer, two convolutional layers, two pooling layers, and fully connected layers (including the output layer), as shown in Figure 6. This study builds upon LeNet-5 architecture, employing the network structure illustrated in Figure 6, and it utilizes the MNIST dataset [31] to validate the proposed methodology.

**Figure 7:**
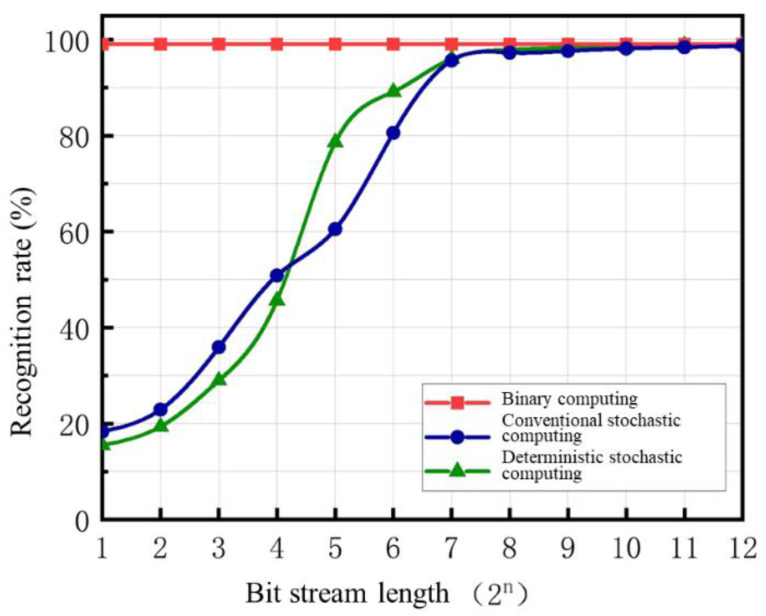
Comparison of Recognition Rates between Traditional Stochastic Computing Networks, Deterministic Stochastic Computing Networks, and Binary Networks under Different Bit. Stream Lengths.

In this network, the multiplication operations across all layers were replaced with both conventional stochastic computing and deterministic stochastic computing, respectively. Addition was performed using an Accumulation Parallel Counter (APC), and the resulting binary values were converted back into stochastic bit streams for computation in the subsequent layer. A comparison was made between the recognition rates of conventional stochastic computing networks with different bit stream lengths, deterministic stochastic computing networks, and binary computing networks. The results are presented in Figure 7. When the bit stream length is less than 32 bits, the resolution of stochastic computing is too low, leading to poor performance for both conventional and deterministic stochastic computing. Additionally, because deterministic stochastic computing can represent one fewer number than conventional stochastic computing for the same bit stream length, its resolution is even lower, resulting in inferior accuracy compared to conventional stochastic computing. The smaller the bit stream length, the more pronounced this resolution-induced difference becomes. However, when the bit stream length is at least 32 bits, the resolution-induced error gradually diminishes. At this point, deterministic stochastic computing achieves a significant accuracy improvement compared to conventional stochastic computing of the same bit stream length. When the stochastic bit stream length reaches 2048 or 4096 bits, the recognition rate of deterministic stochastic computing becomes nearly indistinguishable from that of binary computation.

### 3.3. Co-Optimization Method for Network Training Process

#### 3.3.1. Impact of Different Network Layers on Recognition Rate

The findings in Section 3.2 indicate that deterministic stochastic computing achieves a recognition rate comparable to binary computation when the bit stream length is sufficiently large. However, in practical applications, not all layers of a convolutional neural network require long stochastic bit streams. Dynamically adjusting the bit stream length based on the computational demands and precision requirements of each layer provides a more flexible and efficient approach. For example, hidden layers responsible for complex feature extraction and requiring high computational precision can utilize longer bit streams to enhance data accuracy, thereby capturing and representing feature information more precisely to establish a solid foundation for subsequent computations. Conversely, layers that prioritize computational speed and primarily perform simple feature mapping or rapid data transmission can use shorter bit streams. This approach effectively reduces computational latency, accelerates data processing, and enhances the overall operational efficiency of the network. This method of dynamically adjusting stochastic bit stream length fully leverages the advantages of stochastic computing in neural networks. It is particularly well-suited for parallel computing scenarios, as bit streams of varying lengths can be processed in parallel, maximizing hardware utilization and improving computational parallelism. In terms of reliability computing, the bit stream length can be dynamically adjusted based on the importance of different layers to ensure the computational reliability of critical layers. Additionally, in computationally intensive applications where extreme precision is not essential—such as stochastic encoding in communications and image processing—appropriately tuning the bit stream length can significantly improve computational efficiency while maintaining acceptable accuracy, thereby meeting practical application requirements. In summary, the application of stochastic computing in neural networks—especially the use of different bit stream lengths across different layers—provides an effective strategy to balance hardware efficiency and computational performance.

**Figure 8:**
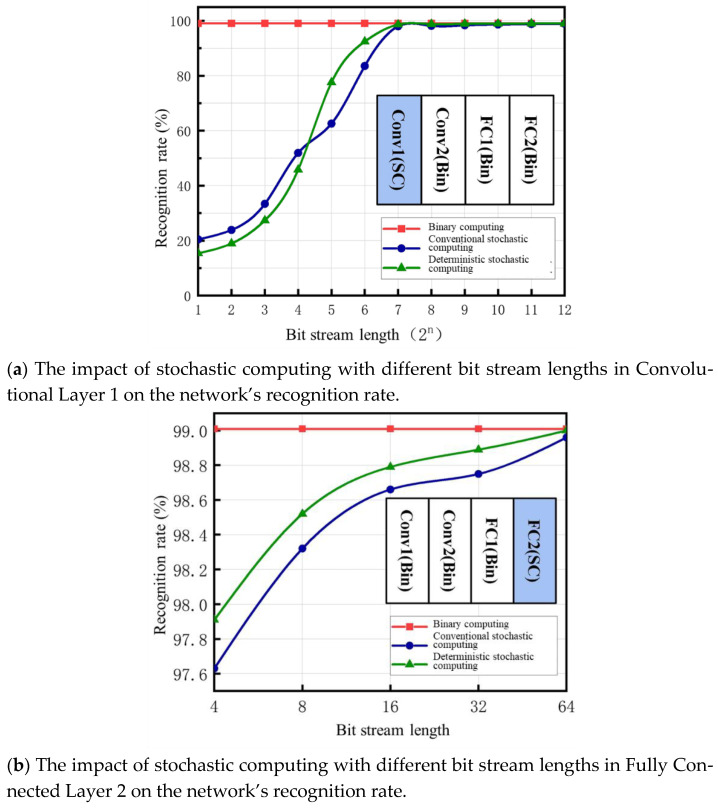
The Impact of Using Stochastic Computing with Different Bit Stream Lengths in Different Network Layers on Network Recognition Rates.

In neural networks, the layers closer to the input layer have a greater impact on the overall accuracy of the network compared to those near the output layer [32]. This is because the layers near the input layer are primarily responsible for extracting raw features, which serve as the foundation for all subsequent layers to perform complex computations and feature fusion. If the features captured near the input layer are inaccurate, the resulting errors will propagate through all subsequent layers, leading to deviations in the network’s feature representation and ultimately affecting the final output. In contrast, the layers near the output layer primarily handle the final classification or regression operations on features that have already been extracted and fused through multiple layers. Their function is more focused on summarizing and consolidating existing information. As a result, even if errors occur near the output layer, their impact is relatively limited to the final few layers. Unlike errors near the input layer, which can cause a cascading effect and lead to a global decline in accuracy, errors near the output layer do not significantly affect the overall network performance. This study investigates the impact of using different stochastic bit stream lengths at various layers of the network on the overall recognition rate, as illustrated in Figure 8. Figure 8a shows the effect of the bit stream length in the first convolutional layer on the recognition rate. In this case, multiplication is performed using a bipolar stochastic computing format, and the resulting values are summed using an APC to obtain binary values, while the subsequent parts of the network operate using binary computation. Figure 8b illustrates the effect of the bit stream length in the output layer (Fully Connected Layer 2) on the recognition rate. Here, only the multiplications in the final fully connected layer are performed using a bipolar stochastic computing format, and the results are summed using an APC to obtain binary values. The rest of the network operates entirely using binary computation.

The results show that when the stochastic bit stream at the output end is reduced to 4 bits, the entire network still achieves a recognition rate of over 97%, regardless of whether traditional stochastic computing encoding or deterministic stochastic computing encoding is used. However, under the deterministic stochastic computing encoding method, the bit stream length at the input end can only be reduced to a minimum of 64 bits, whereas under the traditional stochastic computing encoding method, the minimum allowable bit stream length at the input end is 128 bits. Reducing it further would significantly degrade the network’s recognition rate.

#### 3.3.2. Training Optimization for 2-Bit Deterministic Encoding

As discussed in Section 3.3.1, the length of the stochastic bitstream in layers closer to the input layer has a significantly greater impact on the overall recognition rate of the network compared to layers closer to the output layer. Therefore, to further reduce the power consumption and cost of stochastic computing while improving computational accuracy, this study optimizes and improves the training process of the first convolutional layer in the convolutional neural network.

Since a shifted deterministic bitstream of length n can represent n values, and a PWM-type deterministic bitstream of length n can represent n + 1 values, convolution multiplication can be achieved with only 2-bit deterministic stochastic computing encoding if the values that need to be represented in the network input and the first-layer convolution kernel weights are limited to only two. Table 2 presents the multiplication operations in the first convolutional layer of the network and the corresponding 2-bit deterministic stochastic computing encoding. Following the deterministic encoding method described in Section 3.1, the values are encoded as follows: 1 is represented as 11, −1 as 00, and 0 as 10, enabling convolution multiplication.

To achieve precise multiplication using 2-bit deterministic stochastic computing encoding as shown in Table 2, an additional weight adjustment operation step [33,34] must first be incorporated into the standard training process of the binary network. The objective of this step is to modify the weights so that they approximate a binary distribution upon completion of training. This adaptation enables the replacement of the first convolutional layer with low bit stream length (2-bit) deterministic stochastic computing, with the specific procedure detailed in Algorithm 1. In this algorithm, data represents the input data. n_max denotes the maximum value of the weight scaling factor, which is used to control the range of the scaling factor. N represents the number of training epochs, indicating the number of training iterations for each scaling factor. conv1_weights denotes the weights of the first convolutional layer. Loss represents the loss value, indicating the difference between the model’s predicted output and the actual label target. Min_loss represents the current minimum test loss and is used to record the best model performance. Optimizer refers to the optimizer used to update the model weights. F.nll_loss is the negative log-likelihood loss function, used to calculate the loss between the model output and the target labels. test_loss represents the test loss, indicating the average loss value of the model on the test dataset.
**Algorithm 1** Network Optimization Training ProcessVariables: model instance “model”, weight scaling factor “n”, “train_loader”, test set “test_loader”Input: Model with initial weightsOutput: Model after co-trainingFOR n = 1.0 TO n_maxFOR epoch = 1 TO N:Set model to training modemodel.train()Scale the convolution kernel of the first convolutional layerconv1_weights = model.conv1.weight.data * nRestrict the weights to [−1, 1]conv1_weights = Clamp(conv1_weights, −1, 1)model.conv1.weight.data = conv1_weightsTraining and weight update FOR train_data in train_loader:    data, target = train_data [0], train_data[1]    optimizer.zero_grad()    output = model(data)    loss = F.nll_loss(output, target)    loss.backward()    optimizer.step()    Set model to evaluation mode and evaluate on the test set    model.eval()    test_loss = evaluation(model, test_loader)    Save the model with the best effect of the current scaling factor    IF test_loss < Min_loss:    Save(Model, n)    test_loss = Min_loss

Algorithm 1 presents a multi-stage network optimization training process that dynamically adjusts the scaling factor (n) for the first convolutional layer’s kernels to co-optimize model performance. During each epoch, the algorithm first scales and clips the conv1 weights to the [−1, 1] range before performing standard forward propagation, negative log-likelihood loss computation, and backpropagation. After completing training at each scaling factor stage (from 1.0 to n_max), the model is evaluated on the test set, with only the best-performing version (achieving minimal test loss) being preserved. This design achieves coordinated optimization between constrained convolutional kernel weight adjustment and global model training, ultimately outputting the model trained with the optimal scaling factor configuration.

**Figure 9:**
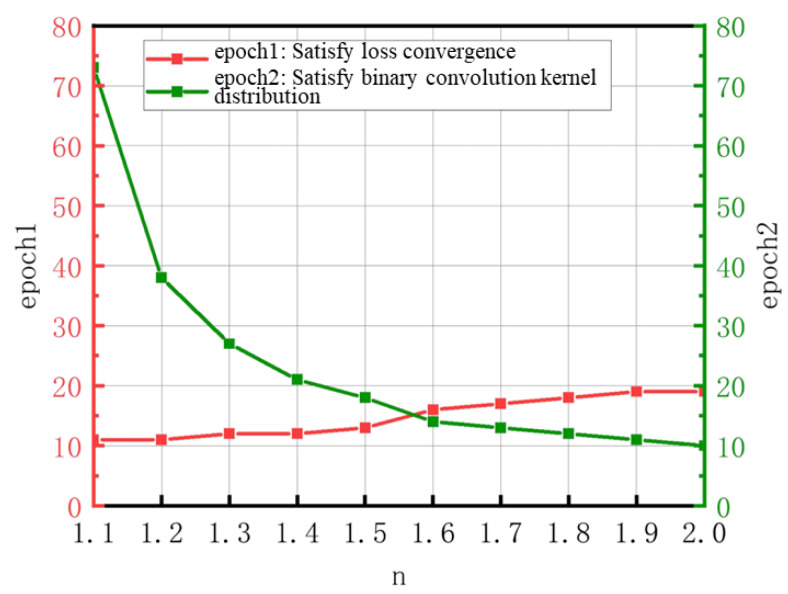
The Number of Epochs Required to Satisfy the Distribution of Binary Convolutional Ker-nels and the Number of Epochs Required for Loss Function Convergence with Different Scaling Factors n.

For the target network, after completing the training process, the following two key conditions should be met: First, after training, the distribution of the convolution kernel weights should align with the expected binary distribution pattern to ensure the network has the specific feature representation and computation characteristics. Second, at the end of the training process, the loss function must converge to a stable state, indicating that the network has found a relatively optimal set of parameters during the optimization process, thereby ensuring the network’s prediction accuracy and stability for input data. Figure 9 compares the number of epochs required to achieve the binary convolution kernel distribution and the number of epochs required for the loss function to converge for different scaling factors, n.

**Figure 10:**
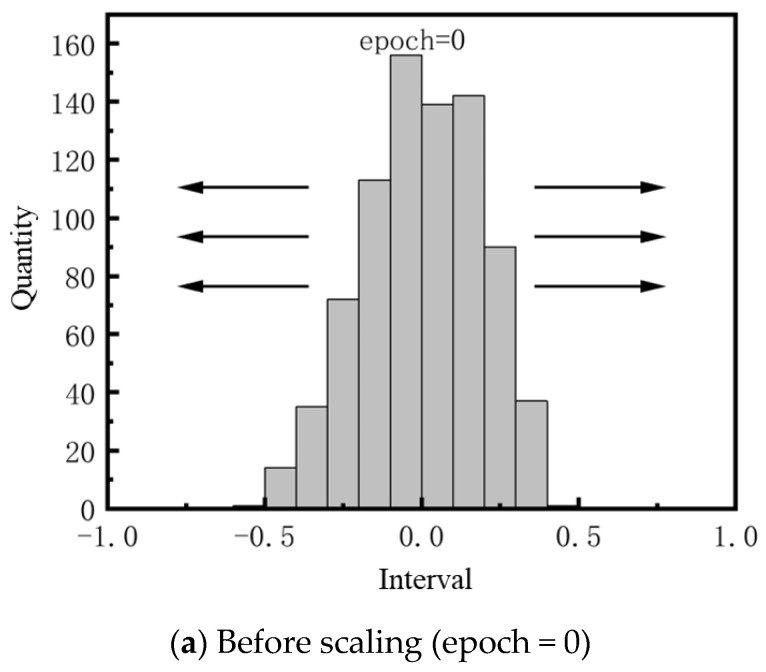

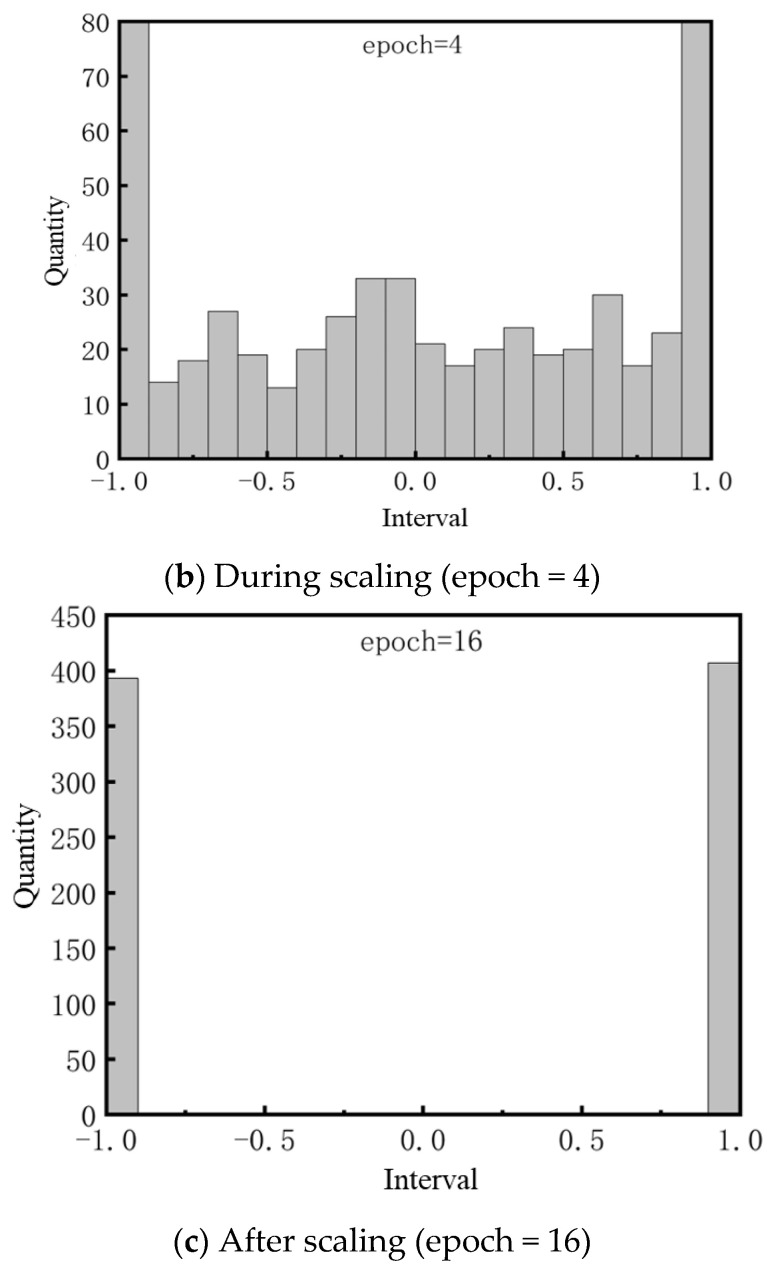
Comparison of Weight Distribution of the First Convolutional Kernel Before and After Scaling.

From the figure, it can be seen that when n = 1.6, the number of epochs required to satisfy both conditions is the smallest, requiring only 16 epochs. Therefore, the final value of n is set to 1.6. Figure 10 shows the comparison of the network’s first convolutional kernel distribution during the training process when the scaling factor is 1.6.

As shown in Figure 10, during the network training process, the distribution of the first layer convolutional kernel weights changed: the weights near 1 and −1 increased continuously, while the weights near 0 decreased. After several rounds of convolutional kernel scaling, the first layer of convolutional kernel weights eventually contained only two values: 1 and −1. The input to the network is the normalized MNIST dataset, which can be considered as containing only two values, 0 and 1. During deterministic encoding, values greater than 0.5 are encoded as 11, while values less than 0.5 are encoded as 10. Since both the input and the weights have only two possible values, the multiplication in the convolution process can be performed using 2-bit deterministic encoding, greatly simplifying the computation while ensuring calculation accuracy.

It should be noted that the proposed method still has limitations in achieving real-time CNN training, making it more suitable for pre-trained model applications rather than meeting the requirements of dynamic learning systems.

## 4. Experiment and Analysis

### 4.1. Comparison of Hardware Consumption and Fault Tolerance Rate

To verify the effectiveness of the proposed near–sensing neural network design method, this section provides a detailed comparison with binary network architectures and traditional stochastic computing methods. The designed convolutional neural network architectures were applied to the first convolutional layer of LeNet-5 and synthesized under a 130 nm process, with an operating frequency set at 100 MHz.

Both the binary and proposed methods were designed as 5 × 5 × 1 convolutional layers for comprehensive evaluation, completing multiply–accumulate operations within a single clock cycle without ping-pong operations. The binary network design was also quantized to 2 bits, using a 2-bit signed multiplier for multiplication. The traditional stochastic computing method employed a linear feedback shift register (LFSR) as the SNG and used XNOR and APC for multiplication and addition, respectively. Since APC was used for accumulation, the summation of multiplication results was independent of correlation. However, the accuracy of XNOR multiplication was correlation-dependent. Thus, in the network structure designed using traditional stochastic computing, an SNG-sharing scheme was adopted, requiring only two SNGs for a 5×5 convolutional window. The traditional stochastic computing scheme implemented designs with 2-bit and 256-bit lengths, with the main difference being the bit width of the SNG and the accumulator. The proposed method follows a design similar to traditional stochastic computing but eliminates the SNG component, thereby reducing SNG-related resource consumption.

Table 3 presents data on area, delay, power consumption, and area-delay product (ADP) for these neural network hardware designs. The delay is calculated as the product of the critical path delay (CPD) and the computation cycle. ADP is a performance and efficiency evaluation metric for circuit design, providing a comprehensive measure of trade-offs between area and speed. A lower ADP value indicates better overall circuit performance and higher efficiency. In Table 3, the binary method is a parallel approach that completes computations in a single clock cycle, so its CPD is equal to the delay. Traditional stochastic computing and the proposed network design operate in serial mode, meaning their computation cycles depend on the length of the stochastic bitstream used. The length of the stochastic bitstream corresponds to the computation cycle.

**Figure 11:**
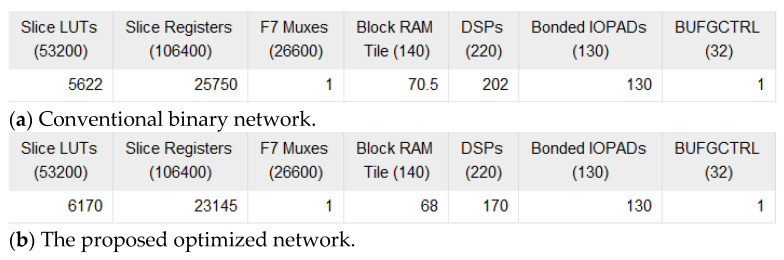
The circuit synthesis results of (**a**) Conventional binary networks (**b**) The proposed optimized network.

From Table 3, it can be seen that compared to traditional stochastic computing schemes, the proposed network design significantly improves in terms of area, delay, and energy efficiency. Compared to the traditional 2-bit stochastic computing scheme, the proposed method reduces area by 44.98%, power consumption by 60.47%, ADP by 44.93%, and increases energy efficiency by 12 times. Compared to the traditional 256-bit stochastic computing scheme, the proposed method reduces area by 82.87% and improves energy efficiency by 1947 times. Compared to the conventional binary design, the proposed method reduces area by 73.56%, power consumption by 57.38%, ADP by 62.87%, and increases energy efficiency by 3.7 times. During the design evaluation, the proposed method eliminates the need for a ramp generator and analog comparator, while both the binary and traditional stochastic computing schemes omit the ADC. Najafi et al. [35] conducted a detailed evaluation of the hardware cost associated with ADCs and PWM wave generation. The area and power consumption required for ADCs are significantly higher than those of ramp generators and analog comparators. Therefore, in practical applications, the energy efficiency improvement achieved by the proposed method maybe even better than the values shown in Table 2. To quantify potential overheads (such as deterministic stochastic computing-to-binary conversion circuits) and evaluate true system-level efficiency, this paper employs Vivado synthesis tools to implement circuit synthesis of the hybrid-encoded neural network using 2-bit deterministic encoding. A comprehensive hardware resource comparison was conducted between conventional binary networks and our proposed hybrid-encoded neural network, with the synthesis results shown in the accompanying Figure 11.

Table 4 compares the fault tolerance results of different network structures under varying bit flip rates, including the binary-form network, as well as networks optimized with traditional 2-bit stochastic computing and deterministic stochastic computing in the first convolutional layer.

Table 5 compares the noise resistance of different network architectures after adding independent and identically distributed Gaussian white noise to the normalized pixel values, where σ is the standard deviation of the Gaussian noise.

For the network recognition rate under varying lighting conditions, two parameters are generally considered: global brightness and local shadow intensity. Among them, the global brightness is represented by Equation (7):

(7) $$I_{out}\left(x,y\right)=I_{in}\left(x,y\right)+\Delta ,\Delta ∼U\left(a,b\right)$$

where Δ is the global brightness offsets, typically ranging from [−0.3, 0.3]. A value of Δ = +0.3 corresponds to overexposure under strong light, while Δ = −0.3 corresponds to underexposure under weak light. Table 6 compares the recognition rates of different network architectures when varying global brightness offsets are applied to the normalized inputs.

The local shadow intensity is represented by Equation (8):

(8) $$I_{out}=I_{in}\cdot \left(1-\alpha M\left(x,y\right)\right)$$

where *M*(*x*,*y*) is the gradient mask, which defines how the shadow intensity smoothly transitions between different regions. The parameter α denotes the shadow opacity level, and the range of *α* is [0.4, 0.8], corresponding to a transmittance of 60% to 20%.

Table 7 compares the recognition performance of different network architectures when varying local shadow densities are applied to the normalized inputs.

Based on the aforementioned experimental results, it can be concluded that when employing our proposed network optimization method, both the deterministic stochastic computing and conventional stochastic computing implementations demonstrate significantly higher fault tolerance compared to binary computing.

### 4.2. Accuracy Evaluation

**Figure 12:**
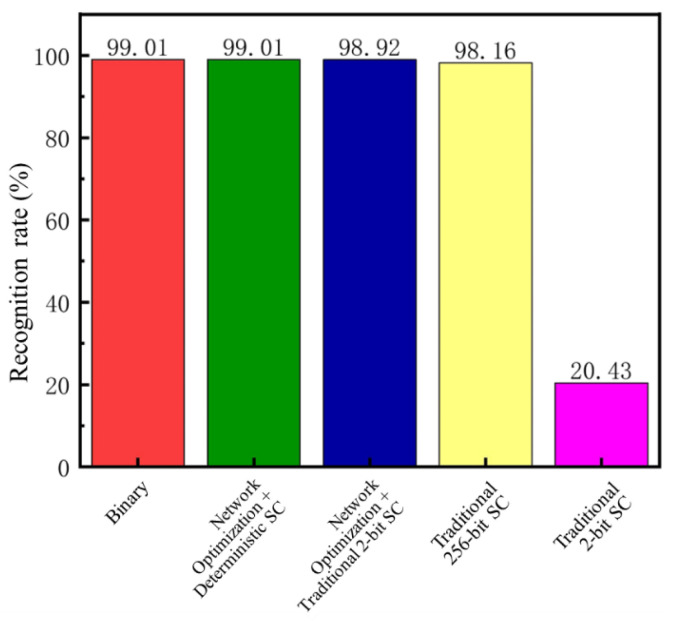
Comparison of Recognition Rates for Different Network Structures.

The structures implemented in Section 3.1 were applied to the first layer of LeNet-5, and the effectiveness of each network design was tested using the MNIST handwritten digit dataset. The binary network and the proposed design used collaboratively optimized trained weights, whereas traditional stochastic computing used non-collaboratively trained weights for testing. The test results are shown in Figure 12.

As seen in Figure 12, after co-training, the network using the proposed optimization method combined with 2-bit deterministic stochastic computing achieved an accuracy of 99.01%, equivalent to that of the binary network. The network using the proposed optimization method combined with traditional 2-bit stochastic computing achieved an accuracy of 98.92%. In contrast, traditional stochastic computing, when using non-collaboratively optimized weights, could only achieve an accuracy of 98.16% even with a 256-bit stream length, resulting in a 0.85% accuracy loss. When using a 2-bit stochastic bit stream length, the accuracy loss was even more significant at 78.58%. This accuracy degradation in stochastic computing is primarily due to the use of XNOR for multiplication, which can cause large error fluctuations when both multiplicands approach zero. The proposed collaborative optimization training method effectively mitigates such errors, enabling deterministic stochastic computing to achieve accuracy equivalent to that of the binary network.

## 5. Conclusions and Outlook

In summary, this study significantly enhances the efficiency of stochastic computing in convolutional neural networks for image classification through an innovative encoding approach, improved input data processing, and collaborative optimization of the network training process. The proposed method enables low-cost, high-efficiency convolutional operations under short bit stream inputs, achieving high recognition performance with minimal bit stream length while significantly reducing system latency and power consumption. Compared to traditional stochastic computing networks, the proposed design shortens the bit stream length by 64 times without compromising recognition accuracy, achieving a 99% recognition rate with a 2-bit input. Compared to the conventional 2-bit stochastic computing approach, the proposed design reduces area by 44.98%, power consumption by 60.47%, and improves energy efficiency by 12 times. Compared to the traditional 256-bit stochastic computing scheme, the area is reduced by 82.87%, while energy efficiency increases by 1947 times. Compared to conventional binary designs, the proposed method reduces area by 73.56%, power consumption by 57.38%, and improves energy efficiency by 3.7 times. These comparative results demonstrate that the proposed design offers significant advantages for tasks such as image classification in near-sensor and edge computing environments, providing an effective solution for high-efficiency, low-cost hardware neural networks.

For future research, the applicability of this method could be further explored in more complex scenarios: First, for large-scale datasets like ImageNet, the input RGB data could be preprocessed into single-channel grayscale images and represented with extended bitstream length. This adaptation would theoretically enable our co-optimization approach to be applied to ImageNet-compatible architectures such as AlexNet. Second, in speech recognition applications, the short-time Fourier transform (STFT) spectrum of speech signals can be directly encoded into PWM-type deterministic bitstreams. Leveraging the inherent sparsity of speech spectra, the weights of the first convolutional layer can be constrained to binary values (−1/1) while appropriately increasing bitstream length to achieve speech recognition functionality. Comprehensive evaluation of hardware costs and reliability in practical deployments will facilitate the application of this method in broader edge computing scenarios.

## Figures and Tables

**Table 1:** Comparison of Contributions and Data from Selected Related Works.

Reference	Main Contributions	Performance Improvements
[15]	Joint optimization method for deep stochastic convolutional neural networks	42.22% improvement in computation accuracy
[16]	Efficient deep convolutional neural network inference framework based on stochastic computing	17% reduction in hardware area, 18% reduction in path delay
[17]	Efficient deep neural network accelerator based on stochastic computing	9.47% improvement in classification accuracy on MNIST dataset
[18]	Efficient FPGA-based implementation of deep neural networks using stochastic computing	82% reduction in hardware resource utilization, 2% increase in accuracy
[19]	Hardware spiking neural network system for pairwise spike-timing-dependent plasticity using stochastic computing	58.0% reduction in hardware resource consumption
[20]	Range-extended stochastic computing for neural network accelerators	2-fold reduction in bit stream length, 3.6 times improvement in energy efficiency
[21]	Computing-in-memory architecture for Bayesian neural networks using stochastic computing	93.6% reduction in energy consumption
[22]	Stochastic computing-based artificial neural network architecture with novel unscaled adder	48% to 81% reduction in power consumption, 51% to 92% reduction in area costs

**Table 2:** 2-bit deterministic stochastic computing multiplication.

Multiplication	Deterministic Stochastic Computing	Results
0 × 1	10 × 11	10 01
0 × −1	10 × 00	01 10
1 × 1	11 × 11	11
1 × −1	11 × 00	00

**Table 3:** Comparison of experimental results.

	Area (μm²)	Delay (ns)	Total Power (μW)	Energy (fJ)	Area-Delay Product (μm² × ms)	Energy Efficiency (TOPs/W)
Binary	4481.12	3.33	116.67	388.52	14.92	40.70
Proposed Structure	1167.95	4.74	34.94	165.59	5.54	150.97
Traditional SC (2-bit)	2122.59	4.74	88.38	418.94	10.06	12.59
Traditional SC (256-bit)	6820.10	1218.56	264.64	322485.49	8310.7	0.08

**Table 4:** The Impact of Different Input Bit Flip Rates on Recognition Results.

	1% Bit Flip Rate	5% Bit Flip Rate	10% Bit Flip Rate
Binary	97.69%	52.04%	20.87%
Network Optimization + Traditional 2-bit SC	98.01%	70.48%	41.26%
Network Optimization + 2-bit Deterministic SC	98.43%	72.43%	42.75%

**Table 5:** The Impact of Different Input Noise Intensities σ on Network Recognition Results.

	σ = 0.1	σ = 0.3	σ = 0.5
Binary	97.21%	85.65%	62.91%
Network Optimization + Traditional 2-bit SC	98.49%	92.59%	76.53%
Network Optimization + 2-bit Deterministic SC	98.51%	92.17%	78.44%

**Table 6:** The Impact of Different Global Brightness Offsets on Network Recognition Results.

	Δ = −0.3	Δ = 0	Δ = 0.3
Binary	73.56%	99.01%	70.38%
Network Optimization + Traditional 2-bit SC	95.77%	98.92%	96.02%
Network Optimization + 2-bit Deterministic SC	96.21%	99.01%	96.14%

**Table 7:** The Impact of Different Shadow Opacity Levels on Network Recognition Results.

	α = 0.2	α = 0.5	α = 0.8
Binary	96.51%	82.68%	63.05%
Network Optimization + Traditional 2-bit SC	97.82%	88.63%	80.94%
Network Optimization + 2-bit Deterministic SC	98.33%	90.14%	79.31%

## Data Availability

The data that support the findings of this study are available from the corresponding author upon reasonable request.
